# Mapping tree canopy thermal refugia for birds using biophysical models and LiDAR

**DOI:** 10.1007/s00484-024-02833-z

**Published:** 2024-11-25

**Authors:** Lara H. Strydom, Shannon R. Conradie, Izak P. J. Smit, Michelle Greve, Peter B. Boucher, Andrew B. Davies, Andrew E. McKechnie

**Affiliations:** 1 Department of Zoology and Entomology, University of Pretoria, Private Bag X20, Hatfield, 0028 South Africa; 2 South African Research Chair in Conservation Physiology, South African National Biodiversity Institute, P.O. Box 754, Pretoria, 0001 South Africa; 3 School of Animal, Plant and Environmental Sciences, University of the Witwatersrand, Johannesburg, South Africa; 4 Scientific Services, South African National Parks, Private Bag X402, Skukuza, 1350 South Africa; 5 Department of Plant and Soil Sciences, University of Pretoria, Private Bag X20, Hatfield, 0028 South Africa; 6 Department of Organismic and Evolutionary Biology, Harvard University, Cambridge, MA USA

**Keywords:** Birds, Climate change, Microclimate, Remote sensing, Thermal landscape

## Abstract

Accurately predicting exposure of animals to climate change requires evaluating the effects of warming on the microclimates they occupy. Birds, like many other taxa, make extensive use of cool microsites in vegetation during hot weather. Taking advantage of recent advances in modelling tree canopy microclimates, we combined LiDAR-based individual tree canopy mapping and biophysical modelling to evaluate the current and future availability of cool microsites in a subtropical African savanna landscape. We constructed biophysical models for two common bird species, an ~ 40-g bulbul and an ~ 200-g hornbill, and modelled exposure to conditions under which the body temperature (*T*_b_) of individuals resting in canopies exceeds 42 °C, equivalent to ~ 2 °C above resting thermoneutral *T*_b_. At present, 34.5% of trees taller than 2 m in our 139-ha study site provide microclimates in which resting *T*_b_ remained below 42 °C for both species during our study period. Under a Representative Concentration Pathway 8.5 climate change scenario and assuming no change in vegetation structure, by the end of the Century the availability of microsites characterized by *T*_b_ < 42 °C will decrease to just 0.4% and 3.8% for bulbuls and hornbills, respectively. The proportion of trees in whose canopies bulbuls’ and hornbills’ exposure to *T*_b_ > 42 °C is limited to < 10 d summer^− 1^ will decrease from 98 to 99% currently to 3.0% and 24.3% by end-century, respectively. These findings reveal the magnitude of changes for birds in a savanna thermal landscape under a business-as-usual emissions scenario.

**Supplementary Information:**

The online version contains supplementary material available at 10.1007/s00484-024-02833-z.

## Introduction

Increases in global temperature and the frequency and intensity of extreme weather events such as heatwaves and droughts associated with advancing anthropogenic climate change (IPCC 2021) are causing myriad effects on animals (Bellard et al. 2012; Parmesan and Yohe 2003). Variation among and within species in terms of their relative vulnerabilities to warming arises from intrinsic organismal and extrinsic environmental variables that determine sensitivity and exposure, respectively (Williams et al. 2008). Protected areas, the cornerstone of modern conservation efforts, shield populations from habitat loss and degradation (Gray et al. 2016), but are not exempt from the impacts of climate change (Araújo et al., 2004; Kharouba and Kerr, 2010). South Africa’s network of National Parks, for example, has seen significant warming in most parks over the past five to ten decades, which in many cases has occurred at rates exceeding mainstream predictions for rates of warming (van Wilgen et al. 2016).

In hot environments, increasing air temperatures (*T*_air_) are associated with a suite of negative effects on birds and other animals. During extreme heatwaves, acute heat exposure over time scales of minutes to hours can lead to mortality when the temperature of an animal’s immediate surroundings exceeds its capacity to defend its body temperature (*T*_b_) at sublethal levels via evaporative cooling or when cumulative evaporative water losses exceed dehydration tolerance limits (Finlayson 1932; McKechnie and Wolf 2010; McKechnie et al. 2021). The risks of lethal hyperthermia increase with atmospheric humidity, as high humidity impedes evaporative heat dissipation and reduced heat tolerance limits (Lasiewski et al. 1966; Ratnayake et al. 2019; Freeman et al. 2024). Chronic exposure to sustained hot conditions over time scales of days to weeks is associated with a suite of sublethal fitness costs, including declining body mass and compromised breeding success (du Plessis et al. 2012; van de Ven et al. 2020; Pattinson et al. 2022). These fitness costs of hot periods result from behavioural trade-offs between foraging and thermoregulation, with missed-opportunity costs arising from increases in behaviours such as panting and shade-seeking (Cunningham et al. 2021).

A key determinant of animals’ exposure to hot weather is the heterogeneity of the thermal landscapes they occupy (Bakken 1976, 1989; Sears et al. 2011). Vegetation is an important modulator of microclimates as leaves intercept solar radiation to create shade, as well as reducing in-canopy temperatures through cooling via evapotranspiration (Oke 2002; Walsberg 1985; Wolf and Walsberg 1996). In particular, shady vegetation can provide cool microclimates with reduced exposure to solar heat loads and their associated high operative temperatures (*T*_e_, Bakken 1976; Robinson et al. 1976). Operative temperature is a more accurate representation of the thermal conditions an organism experiences than *T*_air_, as *T*_e_ incorporates radiative, conductive and convective heat fluxes as well as properties of the organism (Bakken and Gates 1975; Bakken 1976). As *T*_e_ can differ between shaded and exposed microsites by > 10 °C for small birds (e.g., Wolf et al. 2000), occupancy of arboreal microsites during extreme heat can reduce the likelihood of lethal hyperthermia or dehydration. For this reason, predicting species exposure and vulnerability requires incorporation of thermal landscape heterogeneity at spatial scales relevant to the species of interest (Carroll et al. 2016; Tomecek et al. 2017).

The last decade has seen the re-emergence of biophysical ecology as a tool for predicting species’ responses to climate change (reviewed by Briscoe et al. 2023). Biophysical models predict heat exchange, body temperature and behaviour over ranges of environmental temperature when parameterized with organismal traits spanning morphology, behaviour and physiology (Kearney and Porter 2009; Riddell et al. 2019; Briscoe et al. 2023). This approach provides the basis of fine-scale modelling of energy and water balance through space and time, permitting detailed evaluation of range limits and life-history bottlenecks under past, present and future conditions (e.g., Kearney et al. 2016; Mathewson et al. 2017). Although biophysical modelling tools such as the NicheMapR package (Kearney and Porter 2016) can predict microclimates at fine spatial scales, the accuracy of predictions can be refined further by incorporating detailed vegetation structure characteristics (Briscoe et al. 2023). One potential source of such information is high-resolution vegetation mapping using airborne Light Detection and Ranging (LiDAR; Lohani and Ghosh 2017; Nagendra et al. 2013; Davies and Asner 2014). Linking biophysical modelling with LiDAR-based vegetation mapping potentially provides the basis for high-resolution characterisation of thermal landscapes for arboreal animals across large areas, including the incorporation of species-specific thermal physiology into evaluations of habitat suitability at fine spatial scales.

To evaluate the incorporation of LiDAR-based vegetation mapping into biophysical models of avian exposure to lethal effects of extreme heat events, we modelled the diurnal microclimates experienced by different-sized birds resting within tree canopies under current and likely future climate for a 139-ha study site in southern Kruger National Park, South Africa. Our approach involved several steps. First, we used LiDAR data to quantify the height and structural characteristics of every tree with a height > 2 m in the study site. Second, we built a biophysical model using the NicheMapR package and validated predictions before modelling within-canopy microclimates and using these data as input for biophysical models for two representative bird species common in the area, the ~ 40-g dark-capped bulbul (*Pycnonotus tricolor*; hereafter, bulbul) and the ~ 200-g southern yellow-billed hornbill (*Tockus leucomelas*; hereafter, hornbill). We selected these species because of their 5-fold range of body mass and the availability of data on their thermal physiology during heat exposure (van Jaarsveld et al. 2021; Freeman et al. 2022). Finally, we modelled exposure of bulbuls and hornbills to in-canopy microclimate associated with maximum *T*_b_ of perched, resting individuals of each species exceeding a threshold value of *T*_b_ = 42 °C under current and anticipated future climates. The overall aim of the study was to quantify the role of vegetation in buffering birds and other small animals from potentially lethal conditions during heatwaves in a mesic southern African savanna.

## Materials and methods

### Study site

The study was conducted in the Nkuhlu herbivore exclosure (24°58′S, 31°46′E), a 139-ha fenced area inaccessible to meso- and megafauna located on the northern bank of the Sabie River approximately 25 km east of Skukuza in the Kruger National Park (KNP), South Africa (Supplementary Material Figure S1). The area has a summer rainfall climate (Williams et al. 2009) with mean annual precipitation of 550 ± 160 mm (Majozi et al. 2017), an average daily minimum *T*_air_ ranging between 5.6 °C (June) and 20.6 °C (January) and daily maximum *T*_air_ ranging between 25.9 °C (June and July) and 32.6 °C (January; Venter et al. 2003). The exclosure was selected as the study site to reduce the risk of wildlife damaging installed equipment, although woody cover is higher than in the surrounding landscape where it is reduced by herbivores (especially African bush elephants *Loxodonta africana*; Asner and Levick 2012).

The topography of the Nkuhlu exclosure includes a hillcrest, characterised by shallow, sandy soil and foot slopes that are typified by deep, sodic soil (Khomo and Rogers 2005). Woody species common on the sodic foot slopes include *Vachellia grandicornuta*,* Euclea divinorum*, *Spirostachys africana* and *Pappea capensis*, whereas the most common species on the sandy crest are *Dichrostachys cinerea*, *Senegalia nigrescens*,* V. exuvialis*, *Combretum apiculatum*, *C. heroense*, *C. zeyheri*,* Grewia flavescens* and *G. bicolor*, *Lannea schweinfurthii*, *Rhigozum zambesiacum*, *Ormocarpum trichoparum* and *Philenoptera violacea* (Siebert and Eckhardt 2008).

### Vegetation structural mapping

We used LiDAR data collected during January and February 2020 using a rotary-wing Unoccupied Aerial Vehicle (UAV; DJI M600 Pro, DJI, Shenzhen, China). Multiple flights of ~ 15 min each were flown at 8 m s^− 1^ with parallel flight lines at 100 m above ground level following the methods of Boucher et al. (2023). The LiDAR data were collected using the Harvard Animal-Landscape Observatory (HALO; Boucher et al. 2023) sensor package, which contains a Riegl VUX-1 LR (Riegl Laser Measurement Systems, Horn, Austria) laser scanner with a 600 kHz pulse rate. LiDAR data obtaining using the HALO sensor package have been validated previously in KNP (Singh et al. 2023). For the purposes of this study, LiDAR was used to map individual trees > 2 m in height within the Nkuhlu exclosure. LiDAR data were used to extract maximum height and average density values (measured as plant area index following Boucher et al. 2023) for individual trees for model validation and subsequent modelling.

LiDAR point clouds from each flight line were matched together and classified into ground, vegetation, or noise points using the Terrasolid software suite (Axelsson 2000). Following the removal of noise points, the average point density was ~ 150 points m^− 2^. The height above ground was computed for each point in the cloud based on the point’s vertical distance to a triangulated surface model of ground points. Then, the maximum heights of the first return points were rasterized to generate canopy height models (CHMs) at 0.5-m spatial resolution. Canopy density values were calculated for each tree following the workflow in Lidar-Notebooks (Boucher 2023). The foliage density of each tree canopy was computed using the weighted ratio of points in the upper canopy to the points in the rest of the tree, following Boucher et al. (2023).

Trees were segmented from the rasterized CHM following a series of three steps, all performed in R software version 4.0.4 (R Core Team 2021) using the *rLiDAR* (Silva et al. 2017) and *ForestTools* (Plowright and Roussel 2018) packages. The first step was identifying individual treetops, delineated by tree height. This task was accomplished using a window function and variable window function (Popescu and Wynne 2004). The window functions scan the CHM for the highest cells within the set window, and these cells are then tagged as treetops. The second step was to delineate individual tree crowns using a watershed function (Beucher and Meyer 2018) from which individual tree canopy area was calculated for all trees higher than 2 m within the exclosure.

### NicheMapR model and validation of black bulb temperatures

We deployed 34 black bulbs each consisting of a copper sphere painted matt black with a miniature temperature logger mounted in the centre, recording *T*_e_ every 20 min during the austral summer of 2021–2022. The black bulbs were placed in multiple locations across the study site, including exposed and shaded locations in the canopies of several common tree species. The trees were selected to represent the range of available heights (2–18 m) and canopy densities. Further details of black bulb construction and deployment are provided in the Supplementary Materials. The black bulb *T*_e_ data collected in this way were then compared to the outputs of a biophysical model constructed using the NicheMapR package (Kearney and Porter 2016). We used five sub-models implemented in the R programming environment, namely (1) NicheMapR’s base microclimate model, (2) the microclima package (Maclean et al. 2019), (3) the microclimc package (Maclean and Klinges 2021), (4) NicheMapR’s ectotherm model (Kearney and Porter 2020) and (5) NicheMapR’s endotherm model (Kearney et al. 2021). The NicheMapR base microclimate model used the micro_ncep function along with site specific inputs (e.g. location, elevation, slope and aspect) and user-defined inputs (e.g., height of the organisms and maximum percentage shade values) to predict a set of microclimate conditions for a specified location (Kearney and Porter 2016). The NicheMapR package together with the microclima package used NCEP-DOE Reanalysis 2 (Kanamitsu et al. 2002) to obtain historical weather data, which were then downscaled to 30-m^2^ resolution. To quantify air temperature (*T*_air_) in the exclosure plot and to validate the base microclimate model, a Davis Vantage Pro 2 weather station (Davis Instruments, Hayward, USA) was positioned away from tree cover in an open area of the exclosure (24°59’20.9"S 31°46’31.2"E) and calibrated against a mercury-in-glass thermometer with accuracy traceable to the National Institute of Standards and Technology (USA). The distribution of daily maximum *T*_air_ predicted by the base microclimate model did not differ significantly from the weather station data (Kolmogorov-Smirnov, D = 0.143, *p* > 0.05), with predicted numbers of days (d) per 2 °C interval in maximum *T*_air_ accurate to 0.5 ± 3.9 d (range: -4.0 d – 7 d; Supplementary Figure S2).

**Fig. 1:**
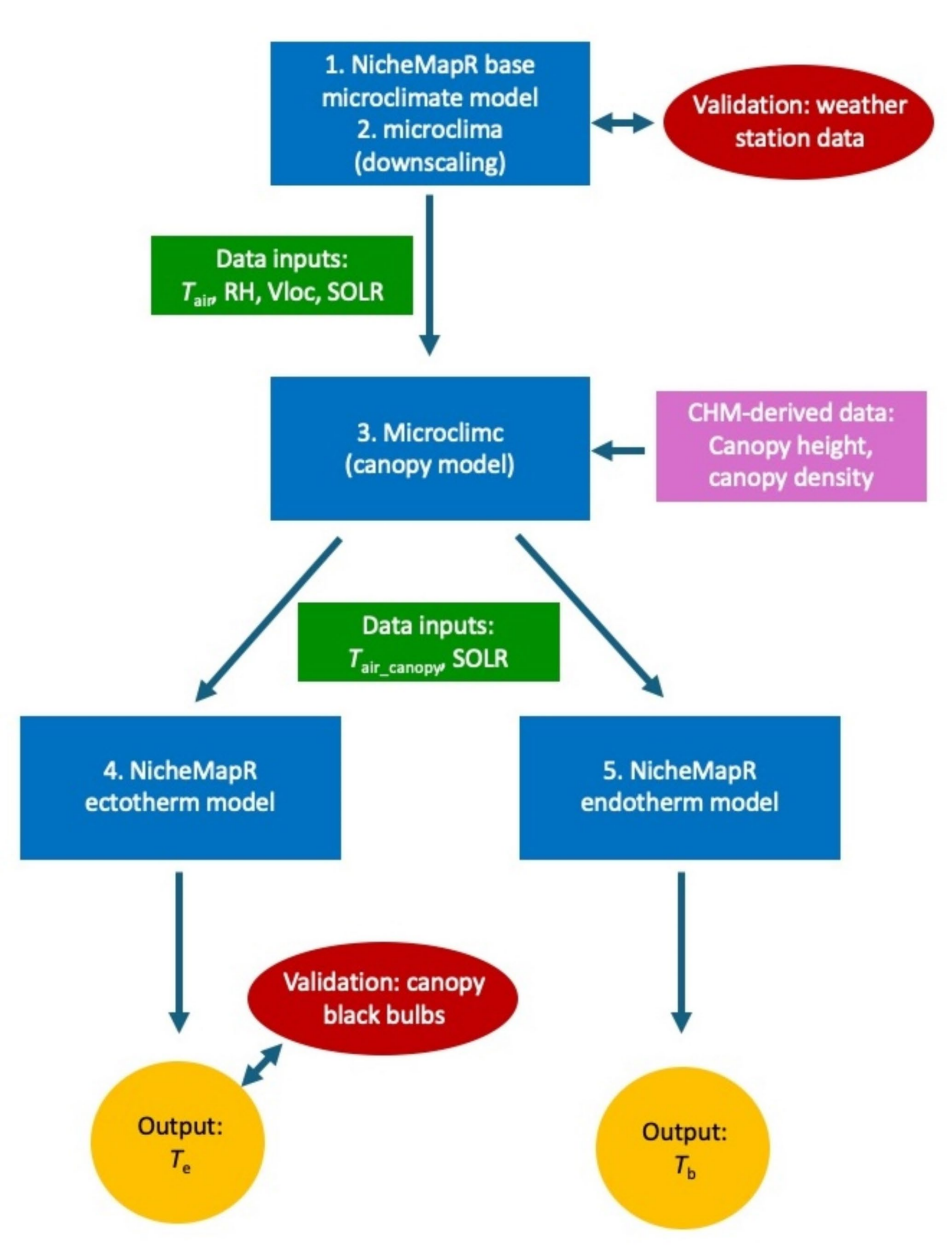
Schematic representation of the process of determining final model operative temperature (*T*_e_) or avian body temperature (*T*_b_) outputs, with the various sub-models incorporated numbered as in the text. Inputs from the base microclimate model included air temperature (*T*_air_), relative humidity (RH), wind speed (Vloc) and incoming solar radiation (SOLR). Data derived from the Canopy Height Model (CHM) included canopy height and density. Inputs from the canopy model to the ectotherm and endotherm models included air temperature within the canopy (*T*_air_canopy_) and SOLR. Validations are indicated using red ovals, with more details provided in the Supplementary Materials

To predict the *T*_e_ of black bulbs deployed in canopies, we used the NicheMapR ectotherm model (Kearney et al. 2020) parameterised for the black bulbs we used, with microclimatic outputs from the base microclimate model used to predict the body temperature experienced by a model ectotherm. The model assumed that the bulbs were filled with tissue, however the conductivity of the flesh was set to 0.025 W m^− 1^ °C^− 1^, similar to the thermal conductivity of air (Graczykowski et al. 2017). We also used the microclimc package (Maclean and Klinges 2021), hereafter referred to as the canopy model, to model *T*_air_ and solar radiation within canopies based on canopy characteristics using input variables derived from the CHM and including individual tree canopy height and density, maximum canopy height and mid-canopy height. In terms of microclimate inputs, *T*_air_, relative humidity (RH), wind speed (VLOC) and incoming solar radiation (SOLR) at the specified heights from the base microclimate model were used as inputs. The microclimate conditions derived from the canopy model were then used as inputs for the ectotherm model to predict black bulb *T*_e_ within canopies (Fig. 1). These *T*_e_ outputs were then validated by comparing the frequency distributions of daily maximum *T*_e_ and *T*_emax_ (Supplementary Material). In general, the model adequately predicted these variables, with the distributions of predicted *T*_emax_ differing significantly from measured values for only 3/11 black bulbs (Supplementary Table S2, Figure S4).

### NicheMapR models for two representative bird species

To model avian *T*_b_ experienced in tree canopies, we used an endotherm model (function endoR_devel) of the NicheMapR biophysical modelling package (version 3.1) and the aforementioned canopy model. The endotherm model has previously been validated for southern yellow-billed hornbills, southern pied babblers (*Turdoides bicolor*) and southern fiscals (*Lanius collaris*) under standard metabolic chamber conditions used for respirometry (Conradie et al. 2023). These authors showed high levels of model accuracy compared to empirical thermoregulatory data across a range of air temperatures (*T*_air_ = 25–40 °C). We extended the biophysical model to hornbills and bulbuls occupying tree canopies, assuming model accuracy under respirometry conditions approximates model accuracy under more complex natural conditions. The detailed workings and validation of the model have been described elsewhere (Kearney et al. 2021; Conradie et al. 2023). In brief, the model uses user-specified physiological and morphological traits and comprises subroutines to calculate the metabolic rate necessary to maintain *T*_b_ given conductive, convective, radiative and evaporative heat exchange with the user-specified surrounding environment (Kearney et al. 2021). Specifically, we used museum measurements of body and plumage dimensions (Conradie et al. 2023) and the microclimate conditions derived from the canopy model as input parameters.

The bulbul and hornbill models were then used to predict *T*_e_ experienced in the canopy of each tree identified in the CHM. To do so, we allocated each tree into one of 100 canopy height X density categories based on canopy height (height: 2–3 m, 3–4 m, 4–6 m, 6–8 m, 8–10 m, 10–12 m, 12–14 m, 14–16 m, 16–18 m or 18–20 m) and density (ratios between 0 and 1 in 0.1 increments based on CHM minimum and maximum values). All vegetation lower than 2 m was excluded during the tree segmentation process to ensure that no grasses, shrubs or saplings were included in our analysis. Of the 100 height X density categories, 61 were represented in Nkuhlu. For each of these, maximum *T*_e_ for bulbuls and hornbills was predicted for each day over the austral summers (October to March) of 2021-22 and 2022-23. All thermal mapping and LiDAR visualisation was performed in QGIS (version 3.24.3 Tisler; QGIS Development Team 2022). We then used these *T*_e_ estimates to model the *T*_b_ of resting bulbuls and hornbills and the frequency of exposure to hyperthermia for both species. Bulbuls and hornbills have normothermic daytime *T*_b_ of 40.4 °C (Freeman et al. 2022) and 39.9 °C (van Jaarsveld et al. 2021), respectively, and we considered *T*_b_ > 42 °C indicative of hyperthermia. We selected *T*_b_ = 42 °C as a threshold as (a) this value is ~ 2 °C above thermoneutral resting diurnal *T*_b_ for both species (van Jaarsveld et al. 2021; Freeman et al. 2022) and (b) it is similar to the mean diurnal *T*_b_ (42.1 ± 0.55 °C) of nine species of birds (24–130 g) held in large flight aviaries during mid-summer at a hot, arid site (Thompson et al. 2018). Thus, we consider *T*_b_ = 42 °C a reasonable threshold for *T*_b_ exceeding thermoneutral setpoints for inactive birds perched in trees during the heat of the day. We also modelled *T*_b_ for the two species based on CMIP5 projections for the period 2080–2100, using experiment r1i1p1 and RCP 8.5 scenario of the CCSM4 projection from CMIP (https://cds.climate.copernicus.eu/cdsapp#!/home).

## Results

### Vegetation structure

**Fig. 2:**
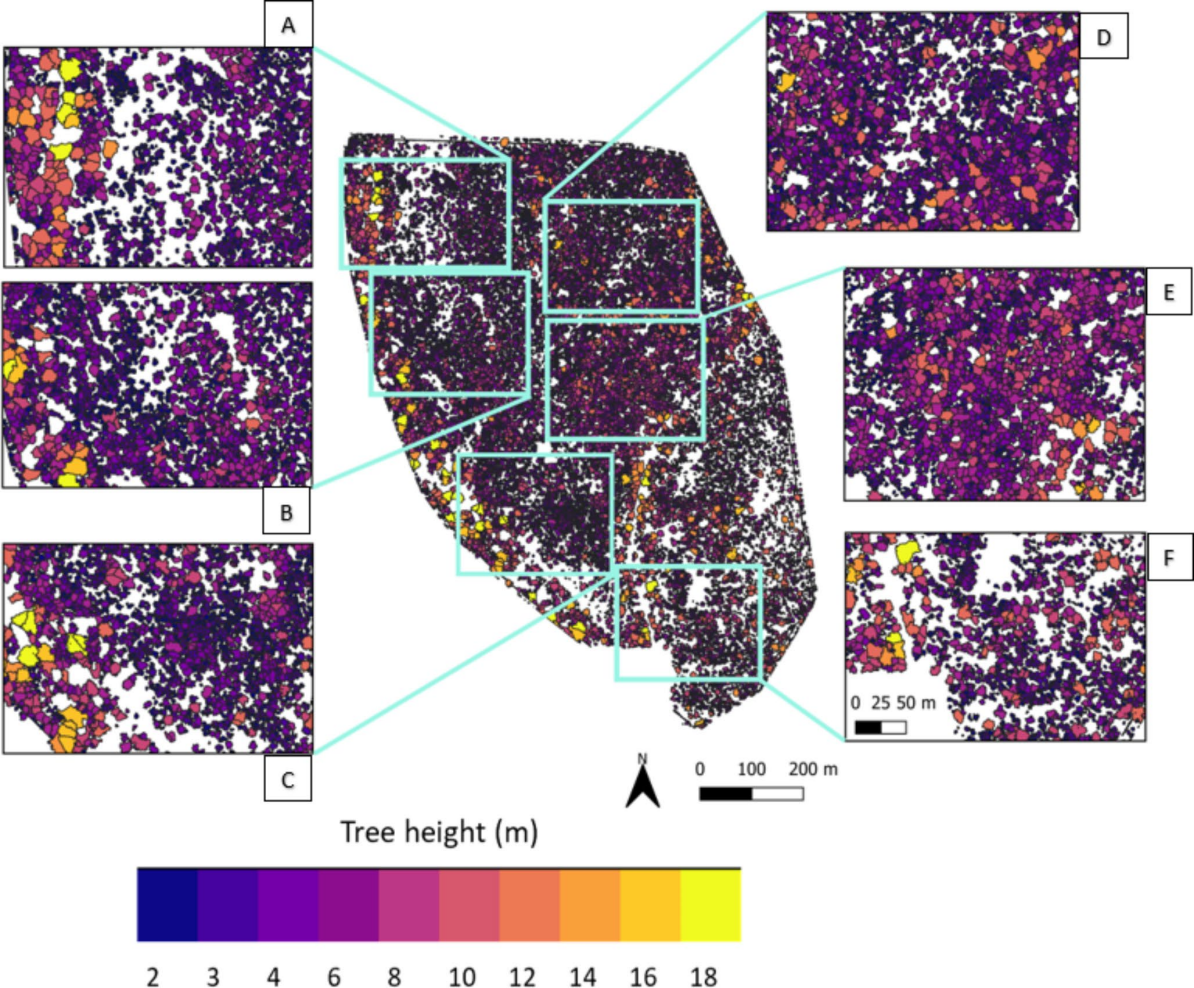
The heights and locations of the trees identified by the LiDAR-derived tree segmentation process in Nkuhlu, Kruger National Park, South Africa. White areas on the map depict bare ground or vegetation < 2 m in height. Insets **A-C** represent riverine and sodic areas, **D** and **E** represent areas with high tree densities and **F** represents an area with lower tree density

The tree segmentation from the LiDAR-derived CHM produced 21,671 individual tree canopies taller than 2 m at the study site (Fig. 2). The height categories of 2–3 m, 3–4 m, and 4–6 m represented 37.2%, 28.2% and 21% of the trees, respectively. A further 8.9% of trees were 6–8 m tall and 4.7% were 8–16 m tall. Very few trees (*n* = 37; 0.2%) were taller than 16 m. Most of the trees (~ 30%) identified by the tree segmentation algorithm had canopy density values between 0.5 and 0.6, and ~ 27% had canopy densities of 0.6–0.7.

### Avian exposure to hyperthermic body temperatures

**Fig. 3:**
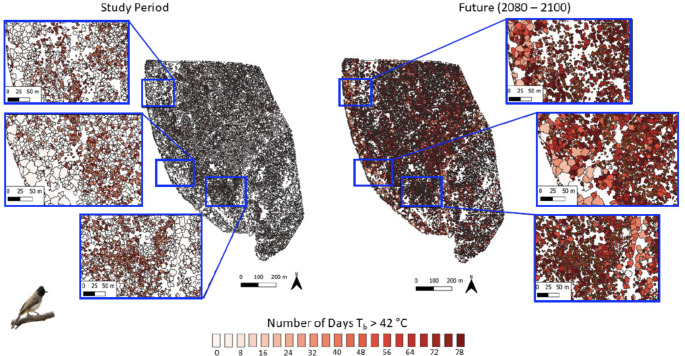
Exposure of dark-capped bulbuls (*Pycnonotus tricolor*; ~40 g) to body temperature (*T*_b_) > 42 °C while resting in vegetation during the austral summers of 2021-22 and 2022-23 (left panel) and under climate predicted for 2080–2100 (right panel) in a 139-ha area of southern Kruger National Park, South Africa, at the scale of individual tree canopies. The colour scale bar shows the total number of days per summer on which a biophysical model predicts bulbuls’ *T*_b_ to exceed 42 °C during the heat of the day

**Fig. 4:**
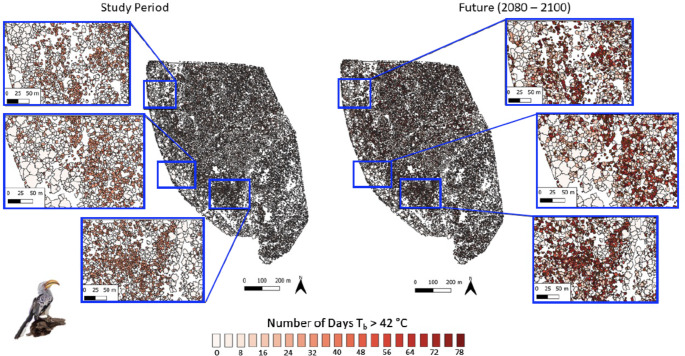
Exposure of southern yellow-billed hornbills (*Tockus leucomelas*; ~200 g) to body temperature (*T*_b_) > 42 °C while resting in vegetation during the austral summers of 2021-22 and 2022-23 (left panel) and under climate predicted for 2080–2100 (right panel) in a 139-ha area of southern Kruger National Park, South Africa, at the scale of individual tree canopies. The colour scale bar shows the total number of days per summer on which a biophysical model predicts hornbill *T*_b_ to exceed 42 °C during the heat of the day

**Fig. 5:**
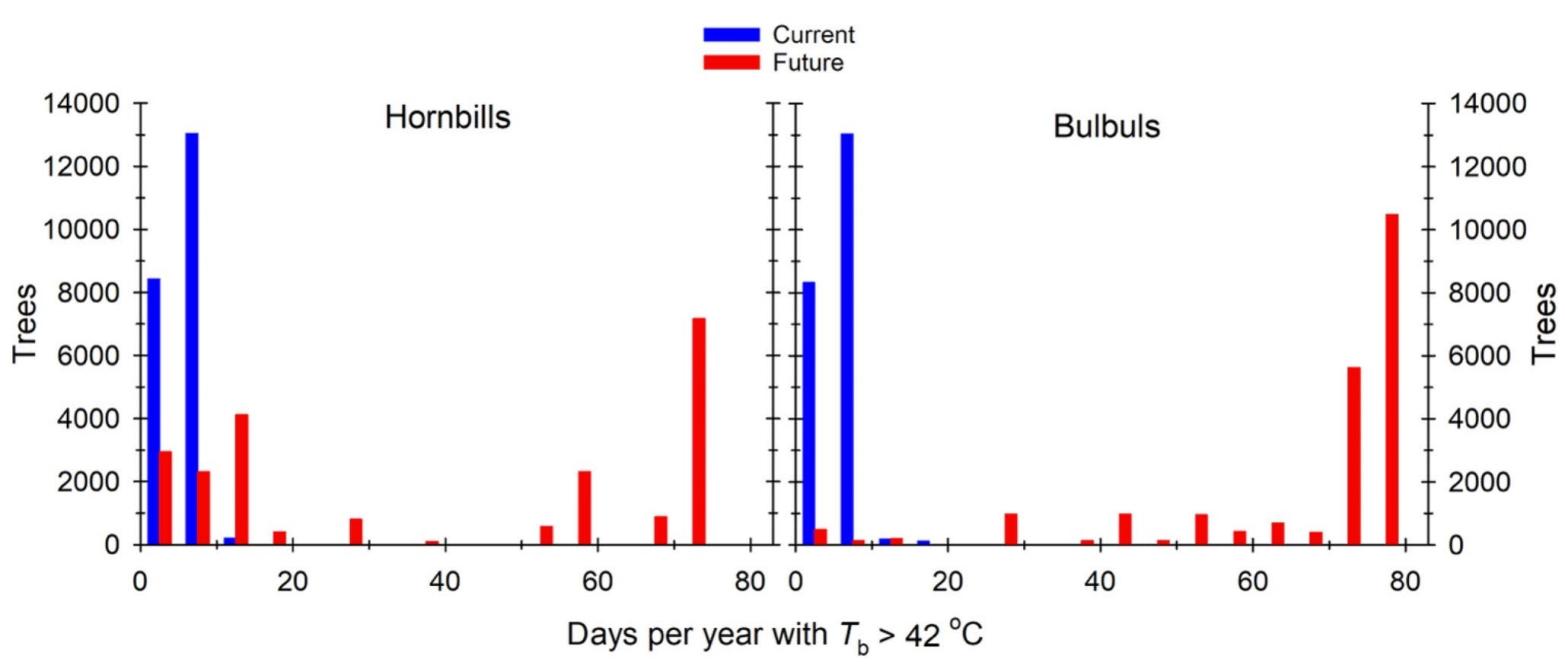
Frequency of current (blue) and future (red; 2080–2100) exposure to days on which body temperature of southern yellow-billed hornbills (*Tockus leucomelas*, left panel) and dark-capped bulbuls (*Pycnonotus tricolor*; right panel) exceeds 42 °C while resting in vegetation in a 139-ha area of southern Kruger National Park, South Africa

Variation in canopy thermal properties was reflected in the frequency at which resting birds are exposed to thermal environments associated with hyperthermic *T*_b_ > 42 °C under current conditions. For dark-capped bulbuls, exposure during the study period varied from < 5 d summer^− 1^ in large trees with dense canopies (Fig. 3, left panel) to > 10 d summer^− 1^ in trees with sparser canopies. The larger southern yellow-billed hornbills experienced quantitatively similar exposure (0–10 d summer^− 1^) to hyperthermic *T*_b_ > 42 °C at present (Fig. 4, left panel). For both species, the canopies of ~ 98.8% of trees at Nkuhlu currently provide microclimates in which resting *T*_b_ exceeds 42 °C on < 10 d summer^− 1^. By the end of the century (assuming no change in vegetation structure), however, exposure for both species will increase substantially, with some canopies exposing birds to *T*_b_ > 42 °C on > 70 d summer^− 1^ (Figs. 3 and 4 right panels). The magnitude of increases will be greater for the 40-g bulbuls compared to the 200-g hornbills (Fig. 5). The percentage of trees at the study site that provide canopy microclimates that will buffer bulbuls from resting *T*_b_ > 42 °C on ≤ 10 d summer^− 1^ will decrease to 3.0%, with 74.3% of trees exposing bulbuls to hyperthermia on > 70 d summer^− 1^ by the end of the Century (Fig. 5). For the hornbills experiencing end-Century climate, 24.3% of tree canopies will limit exposure to ≤ 10 d summer^− 1^ and 33.1% of canopies will shift to exposure levels of 70 > d summer^− 1^.

**Fig. 6:**
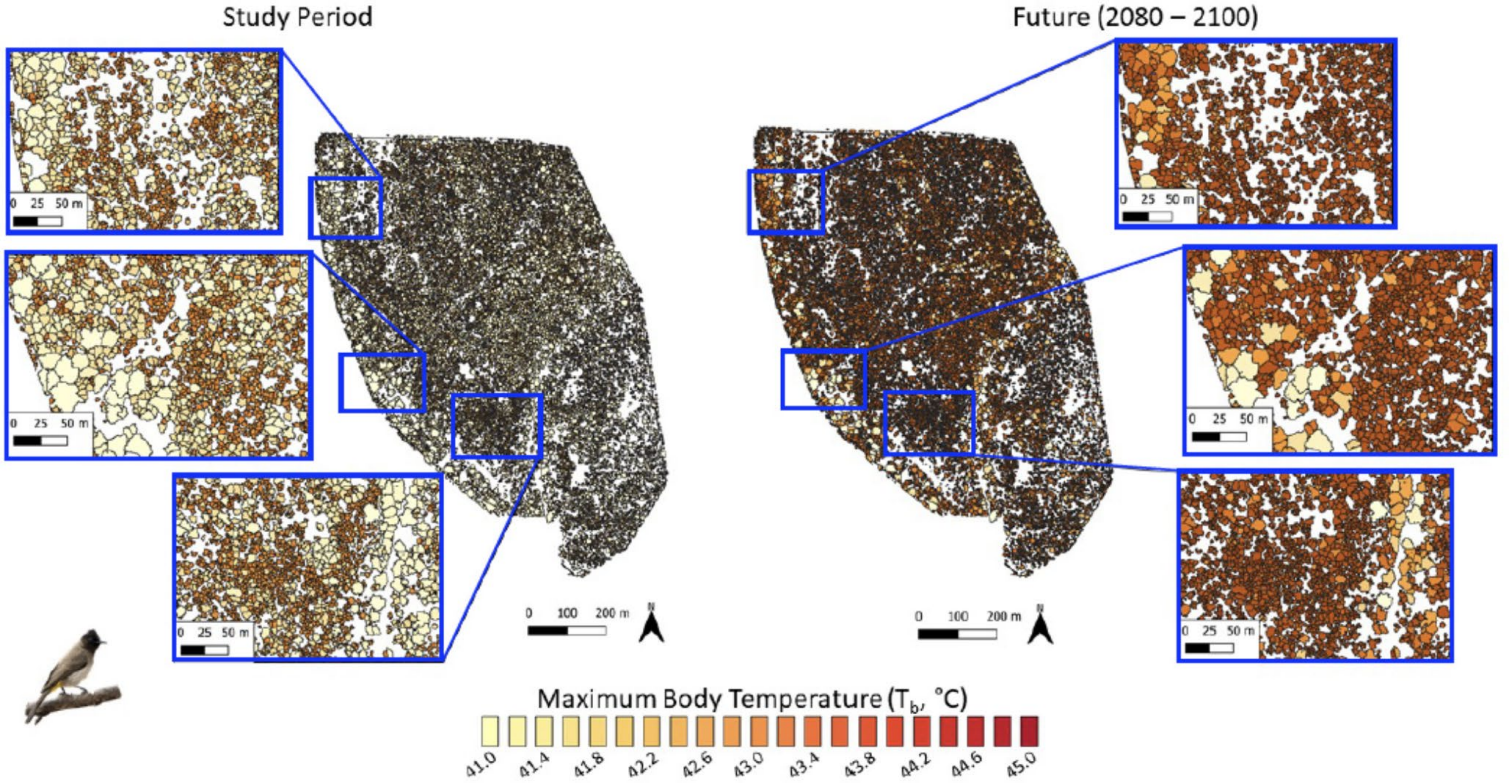
Predicted maximum body temperature (*T*_b_) for dark-capped bulbuls (*Pycnonotus tricolor*; ~40 g) while resting in vegetation during the austral summers of 2021-22 and 2022-23 (left panel) and under climate predicted for 2080–2100 (right panel) in a 139-ha area of southern Kruger National Park, South Africa, at the scale of individual tree canopies

**Fig. 7:**
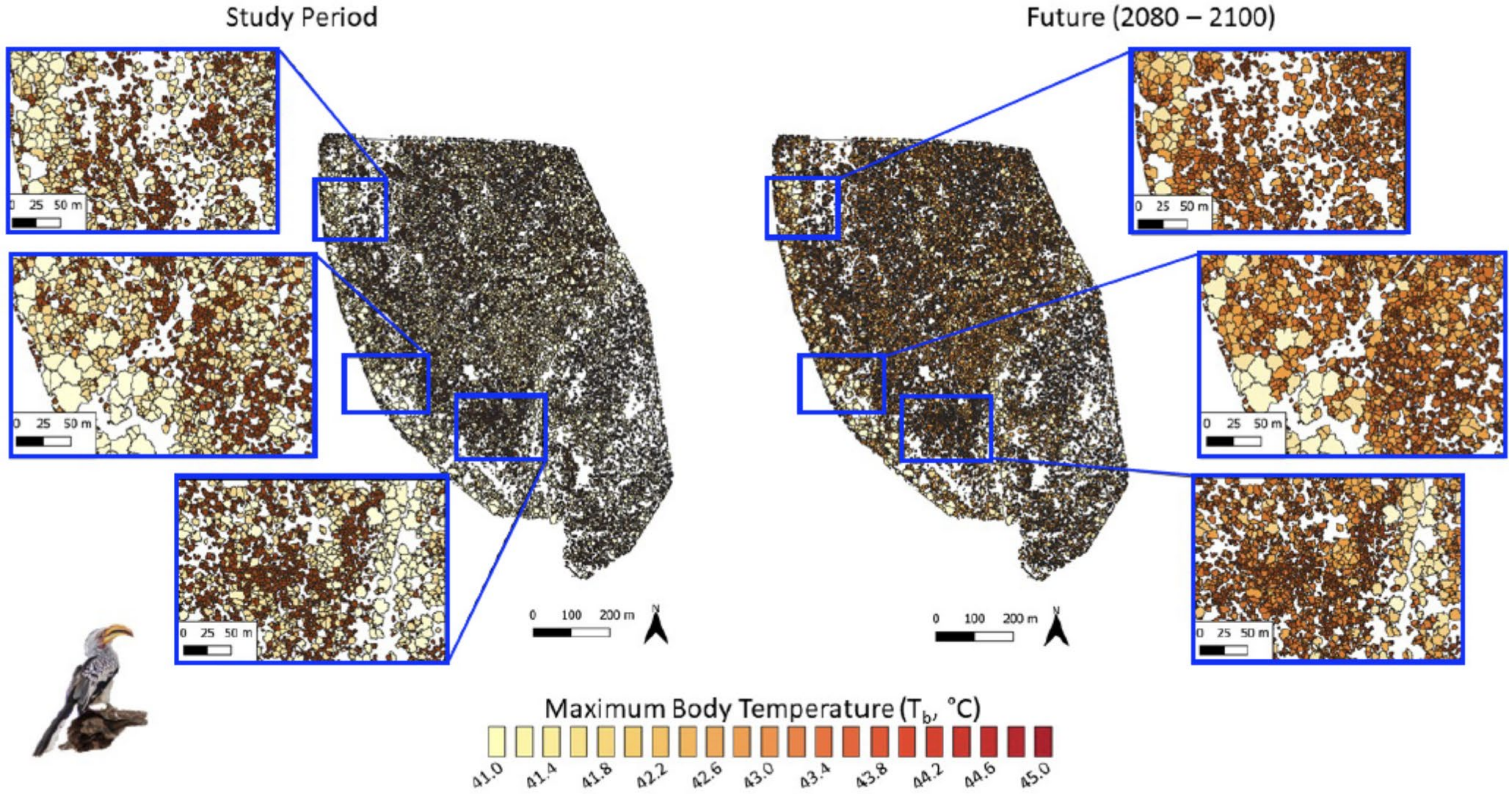
Predicted maximum body temperature (*T*_b_) for southern yellow-billed hornbills (*Tockus leucomelas*; ~200 g) while resting in vegetation during the austral summers of 2021-22 and 2022-23 (left panel) and under climate predicted for 2080–2100 (right panel) in a 139-ha area of southern Kruger National Park, South Africa, at the scale of individual tree canopies

**Fig. 8:**
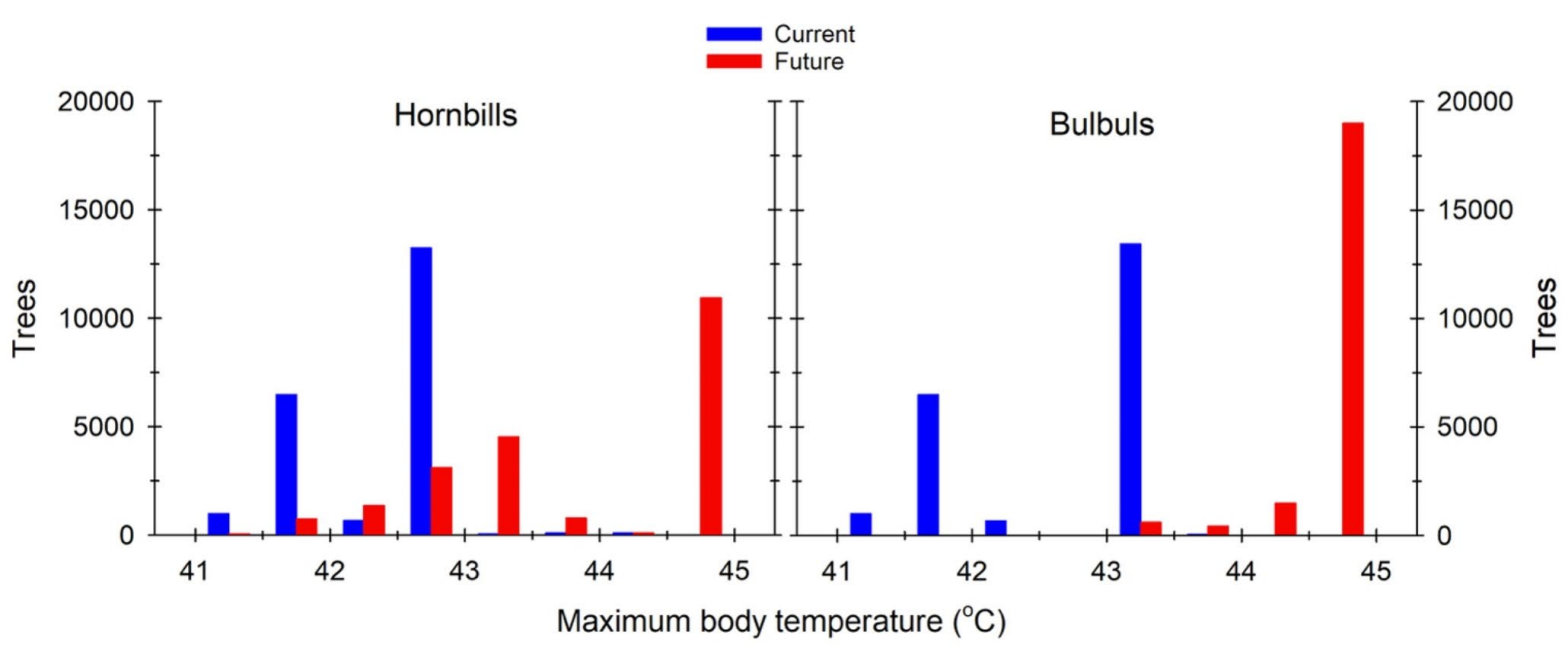
Frequency distributions of maximum body temperatures (*T*_b_) of dark-capped bulbuls (*Pycnonotus tricolor*; left panel) and southern yellow-billed hornbills (*Tockus leucomelas*; right panel) while resting in tree canopies under current (blue) and future (red; 2080–2100) climate. Body temperature categories are binned in 0.5-°C intervals, and frequencies are expressed as the number of trees in which *T*_b_ will not exceed a specific maximum temperature (x-axis) per summer in a 139-ha area of southern Kruger National Park, South Africa

Maximum *T*_b_ experienced by bulbuls and hornbills during summer will also increase substantially (Figs. 6, 7 and 8). At present, 34.5% of trees at Nkuhlu provide canopy microsites within which bulbul and hornbill *T*_b_ remains < 42 °C throughout summer. By the end of the Century, only 0.4% of trees will allow bulbuls to avoid resting *T*_b_ ≥ 42 °C during summer, and *T*_b_ will exceed 44.5 °C in 87.7% of trees. For hornbills, 3.8% of trees will still provide microclimates in which *T*_b_ remains < 42 °C, but most (50.5%) will be associated with *T*_b_ ≥ 44.5 °C (Fig. 8).

## Discussion

Our findings demonstrate that savanna thermal landscapes experienced by birds and other small organisms are highly heterogenous. The marked variation among tree canopies in terms of exposure of resting birds to hyperthermia reveals the importance of dense-canopied tree species as avian thermal refuges during hot weather. Our findings further show how the value of vegetation in providing thermal refuges under future climate varies across bird species. Although both species modelled here will encounter marked declines in the availability of cool microsites in the canopies of large, shady trees by the end of the Century, the magnitude of the decline will be greater for dark-capped bulbuls than southern yellow-billed hornbills (Figs. 5 and 8). Although smaller species may, a priori, be expected to have access to more cool microsites than larger species, we suspect this observation reflects the greater surface area / volume ratios of smaller species and hence higher mass-specific radiative heat gains. Even in tree canopies with near-complete shade, a resting bird still experiences radiative heat gain from surrounding objects with surface temperatures higher than its own, and potentially via reflected solar radiation.

Our analysis focused on hyperthermia exposure during hot weather. However, birds also encounter a risk of dehydration, which can be lethal if cumulative water losses for evaporative cooling exceed dehydration tolerance limits (McKechnie and Wolf 2010; Albright et al. 2017; Conradie et al. 2020). For instance, assuming birds can lose up to 15% of their body mass before lethal dehydration occurs, a bulbul perched in an exposed microsite where *T*_e_ = 48 °C would reach its lethal dehydration limit in 2.5 h (estimated from evaporative water loss measurements for this species by Freeman et al. 2022). In the canopy of a *D. cinerea* (canopy density ratio = 0.617), it would experience *T*_e_ = 46 °C, and would reach 15% body loss in 4.5 h. In contrast, at the same height above ground, but in the denser canopy of a *Kigelia africana* (canopy density ratio = 0.741), it would experience *T*_e_ = 35 °C and only reach its dehydration tolerance limit after 5 h. Dehydration risk scales negatively with body mass, with small birds reaching dehydration tolerance limits more rapidly than larger species for a given *T*_air_ (McKechnie and Wolf 2010). Taken together with our observation that declines in cool microsite availability will be greater for 40-g bulbuls compared to 200-g hornbills, warming may lead to community shifts on account of smaller species being more severely affected by extreme heat events.

The increased hyperthermia and dehydration risks during acute exposure to extreme heat events is only one category of direct impacts of higher *T*_air_. In addition to the impacts of acute heat exposure, rising temperatures are also associated with a suite of sublethal fitness costs associated with missed opportunities because of behavioural trade-offs between foraging and thermoregulation (reviewed by Cunningham et al. 2021). Many of these trade-offs are also dependent on vegetation characteristics and birds’ exposure to high *T*_e_. For instance, arboreal insectivores and frugivores are likely able to forage for longer in the mornings before having to curtail activity during very hot weather compared to species that forage on the ground in exposed locations. Even for the latter foraging guild, however, foraging bouts may be extended if individuals can retreat to deep shade to dissipate heat loads incurred while foraging in the open. The loss of thermally buffered canopy microclimates with advancing climate change may thus affect these behavioural trade-offs if birds can no longer retreat to cool microsites between foraging bouts (du Plessis et al. 2012; Cunningham et al. 2015; van de Ven et al. 2019). Similarly, the impacts of behavioural trade-offs on breeding success and offspring quality (e.g., Cunningham et al. 2013; Bourne et al. 2020; van de Ven et al. 2020) will likely be compounded by the loss of cool canopy microsites. For species that defend territories for part or all of their annual cycle, shade availability and quality will become an increasingly important aspect of territory quality. Overall, our findings reiterate that mesic savanna birds are likely to undergo major declines in coming decades, similar to those predicted for arid-zone species, if climate change predictions hold (Conradie et al. 2019; Ma et al. 2023).

Our study had several limitations. One was that we did not empirically quantify variation in humidity across tree canopies. Humidity increases avian exposure to hyperthermia by impeding evaporative cooling at elevated humidity (Lasiewski et al. 1966; Weathers 1997; Freeman et al. 2024). Elevated humidity is thought to have been a factor in a recent avian mass mortality event in eastern South Africa (McKechnie et al. 2021) and is a predictor of heat-associated mortality among pteropodid fruit bats in Australia (Ratnayake et al. 2019). Although our biophysical models for two bird species incorporated predicted humidity during our study period, future changes in precipitation regimes and humidity could affect hyperthermia risk. A second limitation concerns our assumption that biophysical models were validated using respirometry data under conditions where *T*_air_ ≈ *T*_e_ accurately predict *T*_b_ in more complex natural thermal environments. Validating the models’ predictions here would likely have required taxidermic *T*_e_ mounts of the two species. However, van de Ven et al. (2019) found that 60-mm black bulbs (identical to those we used here) closely matched *T*_e_ measured using hornbill taxidermic mounts, and a NicheMapR model constructed for vervet monkeys (*Chlorocebus pygerythrus*) and validated with metabolic chamber data subsequently accurately predicted *T*_b_ of free-ranging individuals in natural thermal environments (Mathewson et al. 2020). Finally, for a few black bulbs (3/11) our validation revealed significant differences between predicted and measured *T*_emax_ (Supplementary Table S2). Maclean et al. (2021) reported mean absolute errors of ~ 2.8 °C for below-canopy temperatures predicted by micoclimc. The differences between our actual and predicted *T*_e_ should be kept in mind when interpreting our results, and reiterate the scope for refining biophysical models.

Furthermore, we assumed no changes in vegetation structure and quantified end-Century exposure to hyperthermia using current structure. An analysis of future vegetation change in Kruger National Park that incorporated a range of management regimes and climate change scenarios projected the largest overall decreases in net primary production in the Skukuza region (Bunting et al. 2016). However, considerable variation is expected among vegetation size classes, with the area predicted to see large declines in herbaceous and shrub vegetation but increases in tree green leaf, particularly fine-leafed *Vachellia* and *Senegalia* (previously *Acacia*) species (Bunting et al. 2016). Among trees common at Nkuhlu, *Vachellia* spp. tend to have sparse canopies (Supplementary Table S1), whereas the broad-leafed *K. africana*, *S. africana* and *Sclerocarya birrea* are among the species with the coolest canopies. A recent study of *K. africana* in west Africa showed niche conservatism was lacking among subgroups delineated using climate envelopes and consequently this species’ responses to climate will be variable (Yamontche et al. 2024). The distribution of marulas (*S. birrea*) in Eswatini is anticipated to shift out of warmer savanna habitats and into cooler grasslands (Mtsetfwa et al. 2023). If dense-canopied tree species decline at Nkuhlu, our modelling may overestimate the availability of cool canopy microclimates available to birds later this century. Moreover, the higher vegetation density within Nkuhlu compared to surrounding areas because of the exclusion of most herbivores means our models likely overestimate both the current and future availability of buffered tree canopy microsites, making our projections a best-case scenario for this part of KNP.

The role of vegetation in buffered microclimates during hot weather to birds and other arboreal animals has a bearing on several aspects of savanna management, which involves challenges including megaherbivore management (Cook and Henley 2019) fire management (Bond and Midgley 2012) and bush encroachment (O’Connor et al. 2014). For instance, African bush elephants (*Loxodonta africana*) are the primary agent of treefall in KNP and exert an influence on treefall rates up to twice that of fire frequency (Asner and Levick 2012; Asner et al. 2016). Elephant-associated treefall primarily involves trees with heights of 5–9 m and is approximately 6-fold higher compared to experimental herbivore exclosure plots (including Nkuhlu; Asner and Levick 2012), reducing the availability of cool microclimates for birds and other taxa. In protected areas such as KNP, active management of megaherbivore populations is a key element of maintaining thermal landscape heterogeneity and the availability of vegetation providing refuges for birds and other animals during hot weather.

In many regions, savannas are threatened by increases in woody cover associated with several factors (Bond and Midgley 2012; February et al. 2013; Roques et al. 2001). The increase in woody cover in savannas can have negative impacts on biodiversity (Ratajczak et al. 2011), yet the relationship between shrub encroachment and savanna bird assemblages is complex. Bush encroachment generally leads to decreases in bird richness and changes to species assemblages (Sirami et al. 2009; Sheuyange et al. 2005; White et al. 2024), but in some cases the canopy microclimates provided by dense-canopied encroacher species could increase the availability of cool microclimates. The capacity of shrubs to act as refuges from increasing temperatures (Milling et al. 2018) will likely be an important aspect of future studies and management practices for savannas. Current management practices include high intensity fires that effectively reduces woody cover in the short term (Smit et al. 2016; but see Strydom et al. 2023), but frequent and intense fires often result in the loss of tall trees (Shannon et al. 2011; Smit et al. 2016).

By combining LiDAR-derived vegetation structure and biophysical modelling, we were able to quantify current and future microclimates in each of the 21,671 trees taller than 2 m in a 139-area of southern Kruger National Park. Although our study was restricted to one study site, it provides a proof-of-concept that can be applied more widely. Evaluating landscapes in terms of their thermal properties and potential to buffer animals from the negative effects of more frequent and intense heat waves is becoming progressively more important in protected area management. The modelling exercise we present here reveals how the availability of cool microsites for birds and other arboreal animals in savanna habitats will likely decrease in coming decades, with dire consequences for species persistence. Similar to arid-zone birds (Albright et al. 2017; Conradie et al. 2019; Ma et al. 2023), the avifaunas of tropical and subtropical savannas may experience severe declines during the 21st Century as climate change advances.

## Electronic supplementary material

Below is the link to the electronic supplementary material.

Supplementary Material 1

## Data Availability

The datasets generated during and/or analysed during the current study are available from the corresponding author on reasonable request.

## References

[CR1] Albright TP, Mutiibwa D, Gerson AR, Smith EK, Talbot WA, McKechnie AE, Wolf BO (2017) Mapping evaporative water loss in desert passerines reveals an expanding threat of lethal dehydration. Proc Natl Acad Sci 114(9):2283–2288 28193891 10.1073/pnas.1613625114 PMC5338552

[CR2] Asner GP, Levick SR (2012) Landscape-scale effects of herbivores on treefall in African savannas. Ecol Lett 15(11):1211–1217 22863324 10.1111/j.1461-0248.2012.01842.x

[CR3] Asner GP, Vaughn N, Smit IPJ, Levick S (2016) Ecosystem-scale effects of megafauna in African savannas. Ecography 39(2):240–252

[CR4] Axelsson P (2000) DEM generation from laser scanner data using adaptive TIN models. Int Archives Photogrammetry Remote Sens 33(4):110–117

[CR5] Bakken GS (1976) A heat transfer analysis of animals: unifying concepts and the application of metabolism chamber data to field ecology. J Theor Biol 60:337–384 957719 10.1016/0022-5193(76)90063-1

[CR6] Bakken GS (1989) Arboreal perch properties and the operative temperature experienced by small animals. Ecology 70(4):922–930

[CR7] Bakken GS, Gates DM (1975) Heat-transfer analysis of animals: some implications for field ecology, physiology, and evolution. Perspectives of biophysical ecology. Springer, pp 255–290

[CR8] Bellard C, Bertelsmeier C, Leadley P, Thuiller W, Courchamp F (2012) Impacts of climate change on the future of biodiversity. Ecol Lett 15(4):365–377 22257223 10.1111/j.1461-0248.2011.01736.x PMC3880584

[CR9] Beucher S, Meyer F (2018) The morphological approach to segmentation: the watershed transformation. In: Dougherty E (ed) Mathematical morphology in image processing. CRC, pp 433–481

[CR10] Bond WJ, Midgley GF (2012) Carbon dioxide and the uneasy interactions of trees and savannah grasses. Philosophical Trans Royal Soc B: Biol Sci 367(1588):601–612 10.1098/rstb.2011.0182 PMC3248705 22232770

[CR11] Boucher PB (2023) pbb2291/Lidar-Notebooks: Version 1 Release

[CR12] Boucher PB, Hockridge EG, Singh J, Davies AB (2023) Flying high: sampling savanna vegetation with UAV-lidar. Methods Ecol Evol 14(7):1668–1686

[CR13] Bourne AR, Cunningham SJ, Spottiswoode CN, Ridley AR (2020) High temperatures drive offspring mortality in a cooperatively breeding bird. Proceedings of the Royal Society B 287:20201140. 10.1098/rspb.2020.1140 10.1098/rspb.2020.1140 PMC7423658 33043866

[CR14] Briscoe NJ, Morris SD, Mathewson PD, Buckley LB, Jusup M, Levy O, Maclean IM, Pincebourde S, Riddell EA, Roberts JA (2023) Mechanistic forecasts of species responses to climate change: the promise of biophysical ecology. Glob Change Biol 29(6):1451–1470 10.1111/gcb.16557 36515542

[CR15] Bunting EL, Fullman T, Kiker G, Southworth J (2016) Utilization of the SAVANNA model to analyze future patterns of vegetation cover in Kruger National Park under changing climate. Ecol Model 342:147–160

[CR16] Carroll J, Davis C, Fuhlendorf S, Elmore R (2016) Landscape pattern is critical for the moderation of thermal extremes. Ecosphere 7:e01403

[CR19] Conradie SR, Woodborne SM, Cunningham SJ, McKechnie AE (2019) Chronic, sublethal effects of high temperatures will cause severe declines in southern African arid-zone birds during the 21st century. Proc Natl Acad Sci 116(28):14065–14070 31235571 10.1073/pnas.1821312116 PMC6628835

[CR18] Conradie SR, Woodborne S, Wolf BO, Pessato A, Mariette MM, McKechnie AE (2020) Avian mortality risk during heat waves will increase greatly in arid Australia during the 21st Century. Conserv Physiol 8(1):coaa048. 10.1093/conphys/coaa048 32523698 10.1093/conphys/coaa048 PMC7271765

[CR17] Conradie SR, Kearney MR, Wolf BO, Cunningham SJ, Freeman MT, Kemp R, McKechnie AE (2023) An evaluation of a biophysical model for predicting avian thermoregulation in the heat. J Exp Biol 226(15):jeb245066 37470124 10.1242/jeb.245066

[CR20] Cook RM, Henley MD (2019) The management dilemma: removing elephants to save large trees. Koedoe: Afr Protected Area Conserv Sci 61(1):1–12

[CR23] Cunningham SJ, Martin RO, Hojem CL, Hockey PAR (2013) Temperatures in excess of critical thresholds threaten nestling growth and survival in a rapidly-warming arid savanna: a study of common fiscals. PLoS ONE 8(9):e74613 24040296 10.1371/journal.pone.0074613 PMC3767631

[CR22] Cunningham SJ, Martin RO, Hockey PA (2015) Can behaviour buffer the impacts of climate change on an arid-zone bird? Ostrich 86(1–2):119–126

[CR21] Cunningham SJ, Gardner JL, Martin RO (2021) Opportunity costs and the response of birds and mammals to climate warming. Front Ecol Environ 19(5):300–307

[CR24] Davies AB, Asner GP (2014) Advances in animal ecology from 3D-LiDAR ecosystem mapping. Trends Ecol Evol 29(12):681–691 25457158 10.1016/j.tree.2014.10.005

[CR25] du Plessis KL, Martin RO, Hockey PAR, Cunningham SJ, Ridley AR (2012) The costs of keeping cool in a warming world: implications of high temperatures for foraging, thermoregulation and body condition of an arid-zone bird. Glob Change Biol 18:3063–3070 10.1111/j.1365-2486.2012.02778.x 28741828

[CR26] February EC, Higgins SI, Bond WJ, Swemmer L (2013) Influence of competition and rainfall manipulation on the growth responses of savanna trees and grasses. Ecology 94(5):1155–1164 23858655 10.1890/12-0540.1

[CR27] Finlayson HH (1932) Heat in the interior of South Australia – Holocaust of bird-life. S Aus Ornithol 11:158–160

[CR29] Freeman MT, Czenze ZJ, Schoeman K, McKechnie AE (2022) Adaptive variation in the upper limits of avian body temperature. Proceedings of the National Academy of Sciences 119 (26):e2116645119 10.1073/pnas.2116645119 PMC9245658 35727970

[CR28] Freeman MT, Coulson B, Short JC, Ngcamphalala CA, Makola MO, McKechnie AE (2024) Evolution of avian heat tolerance: the role of atmospheric humidity. Ecology 105:e4279. 10.1002/ecy.4279 38501232 10.1002/ecy.4279

[CR30] Graczykowski B, El Sachat A, Reparaz JS, Sledzinska M, Wagner MR, Chavez-Angel E, Wu Y, Volz S, Wu Y, Alzina F (2017) Thermal conductivity and air-mediated losses in periodic porous silicon membranes at high temperatures. Nat Commun 8(1):415 28871197 10.1038/s41467-017-00115-4 PMC5583326

[CR31] Gray CL, Hill SL, Newbold T, Hudson LN, Börger L, Contu S, Hoskins AJ, Ferrier S, Purvis A, Scharlemann JP (2016) Local biodiversity is higher inside than outside terrestrial protected areas worldwide. Nat Commun 7(1):12306 27465407 10.1038/ncomms12306 PMC4974472

[CR32] IPCC (2021) Climate Change 2021: The Physical Science Basis. Contribution of Working Group I to the Sixth Assessment Report of the Intergovernmental Panel on Climate Change

[CR33] Kanamitsu M, Ebisuzaki W, Woollen J, Yang S-K, Hnilo J, Fiorino M, Potter G (2002) NCEP–DOE AMIP-ii reanalysis (r-2). Bull Am MeteorSoc 83(11):1631–1644

[CR36] Kearney MR, Porter W (2009) Mechanistic niche modelling: combining physiological and spatial data to predict species’ ranges. Ecol Lett 12(4):334–350 19292794 10.1111/j.1461-0248.2008.01277.x

[CR90] Kearney MR, Porter WP (2020) NicheMapR–an R package for biophysical modelling: the ectotherm and dynamic energy budget models. Ecography 43(1):85–96

[CR37] Kearney MR, Porter WP (2016) NicheMapR–an R package for biophysical modelling: the microclimate model. Ecography 40(5):664–674

[CR38] Kearney MR, Porter WP, Murphy SA (2016) An estimate of the water budget for the endangered night parrot of Australia under recent and future climates. Clim Change Responses 3(1):14

[CR35] Kearney MR, Gillingham PK, Bramer I, Duffy JP, Maclean IM (2020) A method for computing hourly, historical, terrain-corrected microclimate anywhere on earth. Methods Ecol Evol 11(1):38–43

[CR34] Kearney MR, Briscoe NJ, Mathewson PD, Porter WP (2021) NicheMapR–an R package for biophysical modelling: the endotherm model. Ecography 44(11):1595–1605

[CR39] Khomo L, Rogers K (2005) Proposed mechanism for the origin of sodic patches in Kruger National Park, South Africa. Afr J Ecol 43(1):29–34

[CR40] Lasiewski RC, Acosta AL, Bernstein MH (1966) Evaporative water loss in birds - I. characteristics of the open flow method of determination, and their relation to estimates of thermoregulatory ability. Comp Biochem Physiol 19:445–457

[CR41] Lohani B, Ghosh S (2017) Airborne LiDAR technology: a review of data collection and processing systems. Proc Natl Acad Sci India Sect A: Phys Sci 87:567–579

[CR42] Ma L, Conradie SR, Crawford CL, Gardner AS, Kearney MR, Maclean IM, McKechnie AE, Mi C-R, Senior RA, Wilcove DS (2023) Global patterns of climate change impacts on desert bird communities. Nat Commun 14(1):211 36639376 10.1038/s41467-023-35814-8 PMC9839677

[CR43] Maclean IM, Klinges DH (2021) Microclimc: a mechanistic model of above, below and within-canopy microclimate. Ecol Model 451:109567

[CR89] Maclean IM, Mosedale JR, Bennie JJ (2019) Microclima: an R package for modelling meso- and microclimate. Methods Ecol Evol 10(2):280–290

[CR44] Majozi NP, Mannaerts CM, Ramoelo A, Mathieu R, Mudau AE, Verhoef W (2017) An intercomparison of satellite-based daily evapotranspiration estimates under different eco-climatic regions in South Africa. Remote Sens 9(4):307

[CR45] Mathewson PD, Moyer-Horner L, Beever EA, Briscoe NJ, Kearney M, Yahn JM, Porter WP (2017) Mechanistic variables can enhance predictive models of endotherm distributions: the American pika under current, past, and future climates. Glob Change Biol 23(3):1048–1064 10.1111/gcb.13454 27500587

[CR46] Mathewson PD, Porter WP, Barrett L, Fuller A, Henzi SP, Hetem RS, Young C, McFarland R (2020) Field data confirm the ability of a biophysical model to predict wild primate body temperature. J Therm Biol 94:102754 33292995 10.1016/j.jtherbio.2020.102754

[CR48] McKechnie AE, Wolf BO (2010) Climate change increases the likelihood of catastrophic avian mortality events during extreme heat waves. Biol Lett 6:253–256 19793742 10.1098/rsbl.2009.0702 PMC2865035

[CR47] McKechnie AE, Rushworth IA, Myburgh F, Cunningham SJ (2021) Mortality among birds and bats during an extreme heat event in eastern South Africa. Austral Ecol 46(4):687–691

[CR49] Milling CR, Rachlow JL, Olsoy PJ, Chappell MA, Johnson TR, Forbey JS, Shipley LA, Thornton DH (2018) Habitat structure modifies microclimate: an approach for mapping fine-scale thermal refuge. Methods Ecol Evol 9(6):1648–1657

[CR50] Mtsetfwa FP, Kruger L, McCleery RA (2023) Climate change decouples dominant tree species in African savannas. Sci Rep 13(1):7619 37165034 10.1038/s41598-023-34550-9 PMC10172338

[CR51] Nagendra H, Lucas R, Honrado JP, Jongman RH, Tarantino C, Adamo M, Mairota P (2013) Remote sensing for conservation monitoring: assessing protected areas, habitat extent, habitat condition, species diversity, and threats. Ecol Ind 33:45–59

[CR52] O’Connor TG, Puttick JR, Hoffman MT (2014) Bush encroachment in southern Africa: changes and causes. Afr J Range Forage Sci 31(2):67–88

[CR53] Oke TR (2002) Boundary layer climates. Routledge, Milton Park

[CR55] Parmesan C, Yohe G (2003) A globally coherent fingerprint of climate change impacts across natural systems. Nature 421:37–42 12511946 10.1038/nature01286

[CR56] Pattinson NB, Van De Ven TM, Finnie MJ, Nupen LJ, McKechnie AE, Cunningham SJ (2022) Collapse of breeding success in desert-dwelling hornbills evident within a single decade. Front Ecol Evol 10:842264

[CR57] Plowright A, Roussel J-R (2018) ForestTools: analyzing remotely sensed forest data. R Package Version 02:0

[CR58] Popescu SC, Wynne RH (2004) Seeing the trees in the forest. Photogrammetric Eng Remote Sens 70(5):589–604

[CR54] QGIS Development Team (2022) QGIS Geographic Information System

[CR59] R Core Team (2021) R: a language and environment for statistical computing. Version 4.0.5. R Foundation for Statistical Computing, Vienna

[CR60] Ratajczak Z, Nippert JB, Hartman JC, Ocheltree TW (2011) Positive feedbacks amplify rates of woody encroachment in mesic tallgrass prairie. Ecosphere 2(11):1–14

[CR61] Ratnayake H, Kearney MR, Govekar P, Karoly D, Welbergen JA (2019) Forecasting wildlife die-offs from extreme heat events. Anim Conserv 22:386–395

[CR62] Riddell EA, Iknayan KJ, Wolf BO, Sinervo B, Beissinger SR (2019) Cooling requirements fueled the collapse of a desert bird community from climate change. Proceedings of the National Academy of Sciences:201908791 10.1073/pnas.1908791116 PMC6815107 31570585

[CR63] Robinson DE, Campbell GS, King JR (1976) An evaluation of heat exchange in small birds. J Comp Physiol B 105:153–166

[CR64] Roques K, O’connor T, Watkinson AR (2001) Dynamics of shrub encroachment in an African savanna: relative influences of fire, herbivory, rainfall and density dependence. J Appl Ecol 38(2):268–280

[CR65] Sears MW, Raskin E, Angilletta MJ Jr (2011) The world is not flat: defining relevant thermal landscapes in the context of climate change. Integr Comp Biol 51(5):666–675 21937668 10.1093/icb/icr111

[CR66] Shannon G, Thaker M, Vanak AT, Page BR, Grant R, Slotow R (2011) Relative impacts of elephant and fire on large trees in a savanna ecosystem. Ecosystems 14:1372–1381

[CR67] Sheuyange A, Oba G, Weladji RB (2005) Effects of anthropogenic fire history on savanna vegetation in northeastern Namibia. J Environ Manage 75(3):189–198 15829362 10.1016/j.jenvman.2004.11.004

[CR68] Siebert F, Eckhardt HC (2008) The vegetation and floristics of the Nkhuhlu exclosures, Kruger National Park. Koedoe: African Protected Area Conservation and Science 50 (1):126–144

[CR69] Silva CA, Crookston NL, Hudak AT, Vierling LA, Klauberg C, Silva MCA (2017) Package ‘rLiDAR’. The CRAN project

[CR70] Singh J, Boucher PB, Hockridge EG, Davies AB (2023) Effects of long-term fixed fire regimes on African savanna vegetation biomass, vertical structure and tree stem density. J Appl Ecol 60(7):1223–1238

[CR71] Sirami C, Seymour C, Midgley G, Barnard P (2009) The impact of shrub encroachment on savanna bird diversity from local to regional scale. Divers Distrib 15(6):948–957

[CR72] Smit IPJ, Asner GP, Govender N, Vaughn NR, van Wilgen BW (2016) An examination of the potential efficacy of high-intensity fires for reversing woody encroachment in savannas. J Appl Ecol 53(5):1623–1633

[CR73] Strydom T, Smit IPJ, Govender N, Coetsee C, Singh J, Davies AB, van Wilgen BW (2023) High-intensity fires may have limited medium‐term effectiveness for reversing woody plant encroachment in an African savanna. J Appl Ecol 60(4):661–672

[CR74] Thompson ML, Cunningham SJ, McKechnie AE (2018) Interspecific variation in avian thermoregulatory patterns and heat dissipation behaviours in a subtropical desert. Physiol Behav 188:311–323 29471075 10.1016/j.physbeh.2018.02.029

[CR75] Tomecek JM, Pierce BL, Reyna KS, Peterson MJ (2017) Inadequate thermal refuge constrains landscape habitability for a grassland bird species. PeerJ 5:e3709 28828282 10.7717/peerj.3709 PMC5564388

[CR76] van de Ven T, McKechnie A, Cunningham S (2019) The costs of keeping cool: behavioural trade-offs between foraging and thermoregulation are associated with significant mass losses in an arid-zone bird. Oecologia 191(1):205–215 31420741 10.1007/s00442-019-04486-x

[CR77] van de Ven TM, McKechnie AE, Er S, Cunningham S (2020) High temperatures are associated with substantial reductions in breeding success and offspring quality in an arid-zone bird. Oecologia 193:225–235 32296953 10.1007/s00442-020-04644-6

[CR78] van Jaarsveld B, Bennett NC, Czenze ZJ, Kemp R, van de Ven TM, Cunningham SJ, McKechnie AE (2021) How hornbills handle heat: sex-specific thermoregulation in the southern yellow-billed hornbill. J Exp Biol 224:jeb232777. 10.1242/jeb.232777 33504586 10.1242/jeb.232777

[CR79] van Wilgen NJ, Goodall V, Holness S, Chown SL, McGeoch MA (2016) Rising temperatures and changing rainfall patterns in South Africa’s national parks. Int J Climatol 36(2):706–721

[CR80] Venter FJ, Scholes RJ, Eckhardt HC (2003) The abiotic template and its associated vegetation pattern. In: du Toit JT, Rogers KH, Biggs HC (eds) The Kruger experience: Ecology and management of savanna heterogeneity. Island, Washington DC, pp 83–129

[CR81] Walsberg GE (1985) Physiological consequences of microhabitat selection. In: Cody ML (ed) Habitat selection in birds. Academic, New York, pp 389–413

[CR82] Weathers WW (1997) Energetics and thermoregulation by small passerines of the humid, lowland tropics. Auk 114(3):341–353

[CR83] White J, Stevens N, Fisher J, Reynolds C (2024) Woody plant encroachment drives population declines in 20% of common open ecosystem bird species. Glob Change Biol 30(6):e17340 10.1111/gcb.17340 38840515

[CR85] Williams SE, Shoo LP, Isaac JL, Hoffmann AA, Langham G (2008) Towards an integrated framework for assessing the vulnerability of species to climate change. PLoS Biol 6(12):e325 19108608 10.1371/journal.pbio.0060325 PMC2605927

[CR84] Williams CA, Hanan N, Scholes RJ, Kutsch W (2009) Complexity in water and carbon dioxide fluxes following rain pulses in an African savanna. Oecologia 161:469–480 19582479 10.1007/s00442-009-1405-y PMC2757614

[CR86] Wolf BO, Walsberg GE (1996) Thermal effects of radiation and wind on a small bird and implications for microsite selection. Ecology 77(7):2228–2236

[CR87] Wolf BO, Wooden KM, Walsberg GE (2000) Effects of complex radiative and convective environments on the thermal biology of the white-crowned sparrow (*Zonotrichia leucophrys gambelii*). J Exp Biol 203:803–811 10648222 10.1242/jeb.203.4.803

[CR88] Yamontche C, Houetchegnon T, Gouwakinnou G, Ouinsavi C (2024) Guidelines for sustainable conservation of Kigelia africana based on ecological niche modelling under climate change in Benin, West Africa. Model Earth Syst Environ 10:3359–3373

