# Supplementary material for: Mapping tree canopy thermal refugia for birds using biophysical models and LiDAR
Source: Int J Biometeorol. 2024 Nov 25;69(10):2461–74. doi: 10.1007/s00484-024-02833-z (PMC12540547; doi:10.1007/s00484-024-02833-z)
Supplement: Supplementary file 1 — Supplementary Material 1 [file 484_2024_2833_MOESM1_ESM.docx]

**Supplementary Materials**

Mapping tree canopy thermal refugia for birds using biophysical models and LiDAR

*International Journal of Biometeorology*

Lara H. Strydom, Shannon R. Conradie^,^ Izak P.J. Smit, Michelle Greve, Peter Boucher, Andrew B. Davies, Andrew E. McKechnie^*^

*Corresponding author

Department of Zoology and Entomology, University of Pretoria, Private Bag X20, Hatfield 0028, South Africa

South African Research Chair in Conservation Physiology, South African National Biodiversity Institute, P.O. Box 754, Pretoria 0001, South Africa

Email: [andrew.mckechnie@up.ac.za](mailto:andrew.mckechnie@up.ac.za)

*Validation of base microclimate model*

The base microclimate model predicted maximum (*T*_air_max_), average (T_air_avg_) and minimum (*T*_air_min_) air temperatures recorded by the weather station during October 2021 – May 2022 (Figure S1) with reasonable accuracy. The *T*_air_max_ values predicted by the model did not differ significantly (Kolmogorov-Smirnov, D = 0.143, p>0.05) from those recorded by the weather station, although the base microclimate model tended to overestimate days with *T*_air_max_ ≤ 29 °C and underestimate days on with *T*_air_max_ > 29 °C (Figure S1). The base microclimate model also predicted *T*_air_avg_ values not significantly different to those recorded by the weather station, but *T*_air_min_ values predicted by the model differed significantly (p < 0.05) to those recorded by the weather station.

#### Validation of NicheMapR predictions of in-canopy operative temperatures

To validate the ectotherm model’s predictions of in-canopy operative temperatures (*T*_e_), we compared model outputs to *T*_e_ measured using black bulb thermometers. Each black bulb consisted of a copper sphere of either 30-mm or 60-mm diameter (wall thickness of 0.9 mm) spray-painted matt black, with a miniature temperature logger (Thermochron iButton, model DS1922L, Maxim Integrated Products, San Jose CA, USA) mounted centrally within the sphere and not touching the inner surface. Each iButton was placed in a retainer (iButton Retainer SMD, Maxim Integrated Products) with a wire soldered to each terminal, allowing us to download data and reprogram each iButton without having to remove it from the black bulb. The iButtons were programmed and data downloaded using a modified blue dot reader (DS1402D-DR8, Maxim Integrated Products) and ColdChain Thermodynamics software (Maxim Integrated Products). Once deployed, iButtons recorded temperature at 20-min intervals with 0.065 °C accuracy. Prior to the study, we calibrated a subset of iButtons (n=27) over a range of temperatures in a water bath measured using a mercury-in-glass thermometer with NIST-traceable accuracy. A linear model fitted to actual temperature as a function of measured temperature revealed a maximum error of 0.48 °C. We placed 22 black bulbs in the canopies of 11 tree species at Nkuhlu (Table S1, Figure S2), with one 30-mm and one 60-mm bulb placed within 50 cm of each other in the approximate centre of each tree’s canopy and secured 10-20 cm from a branch using a piece of wire affixed to the inside of each bulb at one end and loosely twisted around the branch at the other. The trees were selected to represent a range of heights (2-18m) and canopy densities within the exclosure Data were collected between October 2021 and April 2022.

The maximum *T*_e_ (*T*_emax_) values recorded by black bulbs varied widely between trees (Figure 6 and Figure 7). Daily maxima varied from ~ 50 °C in *Dichrostachys cinnerea* to ~ 36 °C in *Kigelia africana* (Figure 6 and Figure 7). The biophysical model accurately predicted daily *T*_emax_ values for 7 of 11 study trees (i.e., predicted *T*_emax_ did not differ significantly from values recorded by the small and large bulbs) (Table S2). For the other four trees, the models predicted within a 95% confidence interval (p > 0.05) the T_emax_ recorded by the large bulb of *Philenoptera violacea*, and for the small bulb of *Combretum hereroense* (Table S2). For *Combretum apiculatum* and *Kigelia africana*, the T_emax_ predicted by the biophysical models differed significantly (p < 0.05) from T_emax_ recorded by both the large and small bulbs.

Table S1. Tree species in whose canopies black bulbs were deployed in Nkuhlu exclosure, southern Kruger National Park. The canopy density values (the weighted ratio of points in the upper canopy to point in the rest of the tree) and maximum tree height were derived from canopy height model products.

| Species | Canopy density value | Maximum height of tree canopy (m) | Period deployed |
| --- | --- | --- | --- |
| *Combretum hereroense* | 0.414 | 3.07 | Feb – Apr 22 |
| *Vachellia gerrardii* | 0.471 | 7.02 | Feb – Apr 22 |
| *Combretum apiculatum* | 0.476 | 2.86 | Oct 21 – Apr 22 |
| *Vachellia grandicornuta* | 0.601 | 6.61 | Feb – Apr 22 |
| *Dichrostachys cinerea* | 0.617 | 2.10 | Feb – Apr 22 |
| *Terminalia sericea* | 0.620 | 6.80 | Feb – Apr 22 |
| *Philenoptera violacea* | 0.642 | 8.13 | Oct 21 – Apr 22 |
| *Senegalia nigrescens* | 0.643 | 4.27 | Oct 21 – Apr 22 |
| *Sclerocarya birrea* | 0.676 | 10.27 | Oct 21 – Apr 22 |
| *Spirostachys africana* | 0.688 | 8.41 | Feb – Apr 22 |
| *Kigelia africana* | 0.741 | 14.67 | Oct 21 – Apr 22 |

Table S2. Kolmogorov-Smirnov test (D and p-values) comparing the daily maximum operative temperatures (T_emax_) and daily average operative temperatures (T_eavg_) predicted by the biophysical models and the canopy black bulbs. “L” and “S” indicate large (60 mm) and small (30 mm) bulbs respectively. Predicted and observed values that differed significantly (p < 0.05) are indicated in bold.

| Tree ID | Bulb size | Daily T_emax_ | |
| --- | --- | --- | --- |
|  |  | D | p-value |
| *Vachellia gerardii* | L | 0.214 | 0.152 |
|  | S | 0.232 | 0.098 |
| *Senegalia nigrescens* | L | 0.073 | 0.753 |
|  | S | 0.062 | 0.900 |
| *Terminalia sericea* | L | 0.214 | 0.153 |
|  | S | 0.232 | 0.098 |
| *Dichrostachys cinerea* | L | 0.132 | 0.707 |
|  | S | 0.143 | 0.617 |
| *Sclerocarya birrea* | L | 0.145 | 0.057 |
|  | S | 0.135 | 0.090 |
| *Vachellia grandicornuta* | L | 0.112 | 0.872 |
|  | S | 0.250 | 0.060 |
| *Spirostachys africana* | L | 0.179 | 0.334 |
|  | S | 0.168 | 0.403 |
| *Kigelia africana* | L | 0.217 | **<0.001** |
|  | S | 0.170 | **0.015** |
| *Combretum apiculatum* | L | 0.221 | **<0.001** |
|  | S | 0.161 | **0.025** |
| *Combretum hereroense* | L | 0.358 | **0.001** |
|  | S | 0.252 | 0.056 |
| *Philenoptera violacea* | L | 0.073 | 0.086 |
|  | S | 0.149 | **0.048** |


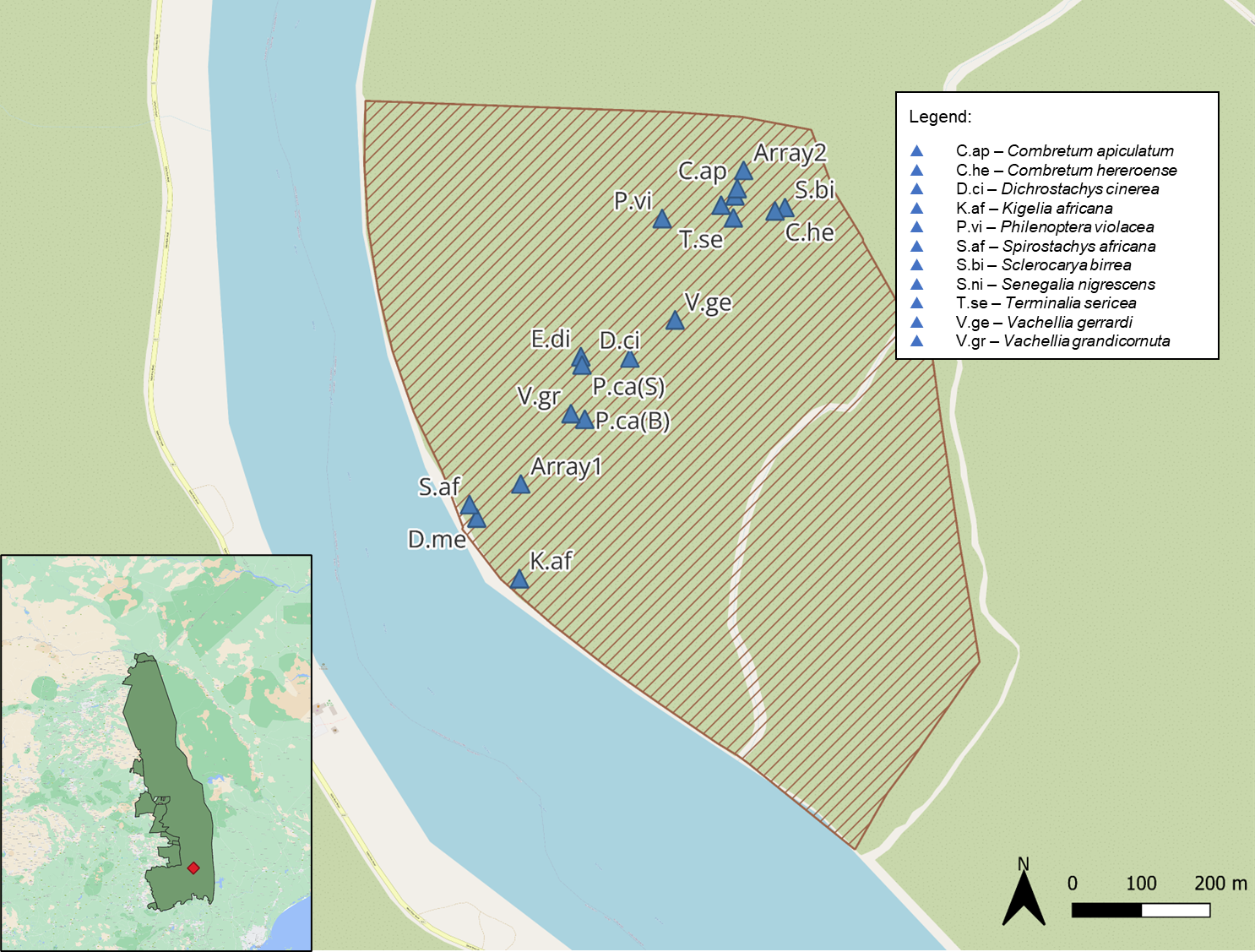


Figure S1. Location of trees in which black bulbs were deployed (blue triangles) to validate operative temperature (*T*_e_) predicted by the *NicheMapR* ectotherm model in Nkuhlu exclosure (brown cross-hatching in main figure; red diamond in inset map) in southern Kruger National Park.

Figure S2. Daily maximum air temperature (*T*_air_max_) values predicted by the base microclimate model (dark grey), compared to corresponding values recorded by a weather station (light grey). For ease of visualisation, the temperatures have been grouped into 2-°C categories from 20 °C to 38 °C, and the x-axis values the midpoint for each. Data were recorded between October 2021 and May 2022.

Figure S3. Daily black bulb maximum operative temperature (*T*_emax_) values recorded by large black bulbs in the canopies of seven tree species for two weeks in February 2022, listed in order of ascending canopy density.

|  |  |
| --- | --- |
|  |  |

Figure S4. Daily maximum operative temperature (*T*_emax_) values recorded by large black bulbs within four tree canopies (light grey) compared to the daily operative *T*_emax_ values predicted by the models (base microclimate, canopy and ectotherm) (dark grey). A (*Sclerocarya birrea*) and B (*Senegalia nigrescens*) show data collected between October 2021 and April 2022; C (*Terminalia sericea*) and D (*Spirostachys africana*) show data collected between February 2022 and April 2022.
